# Male partner involvement in delivery care service and associated factors in Ethiopia: a systematic review and meta-analysis

**DOI:** 10.1186/s12913-024-11993-y

**Published:** 2024-11-26

**Authors:** Chalie Mulugeta, Tadele Emagneneh, Getinet Kumie, Assefa Sisay, Abebaw Alamrew

**Affiliations:** 1 Department of Midwifery, College of Health Science, Woldia University, Woldia City, Ethiopia; 2 Department Of Medical Laboratory Science, College of Health Science, Woldia University, Woldia City, Ethiopia

**Keywords:** Male involvement, Childbirth, Male, Labor, Ethiopia

## Abstract

**Introduction:**

Promoting the involvement of male partners in skilled delivery care is a strategy supported by the World Health Organization (WHO) to improve maternal and infant health outcomes. This systematic review and meta-analysis aimed to estimate the pooled prevalence of male partner involvement in delivery care service in Ethiopia and its contributing factors.

**Methods:**

We retrieved observational studies conducted in Ethiopia from PubMed, Google Scholar, Embase, Cochrane Library, Hinari, and Mednar using Boolean search terms. The Newcastle Ottawa 2016 Critical Appraisal Checklist assessed the methodological quality of the studies. Publication bias was evaluated with a funnel plot and Egger’s test, and heterogeneity was checked using the I-squared test. Data were extracted into Microsoft Excel and analyzed using Stata 11 software.

**Results:**

10 articles with 5,307 participants were included for analysis. The pooled prevalence of male partner involvement in delivery care service was 52.99% (95% CI: 40.63–65.35). Paternal secondary education and above (OR 1.99, 95% CI: 1.69, 2.30), paternal knowledge (OR 3.129, 95% CI: 1.901, 4.356), partner attitude (OR 2.39, 95% CI: 1.45–3.34), having ANC accompanying experience (OR 8.09, 95% CI: 3.14, 19.32), and urban residence (OR 2.12, 95% CI: 1.61, 2.64) were significantly associated with male partner involvement in delivery care service in Ethiopia.

**Conclusion:**

This study found that more than half of male partners in Ethiopia were involved in delivery care services. The key contributing factors for male partner involvement in delivery care services were paternal secondary education and above, paternal knowledge, partner attitude, ANC accompanying experience, and urban residence. Campaigns should be organized to improve knowledge, attitude, and effectively recognize men’s involvement in skilled birth care.

**Supplementary Information:**

The online version contains supplementary material available at 10.1186/s12913-024-11993-y.

## Introduction

In 2020, around 287,000 women died globally from pregnancy-related causes, with 99% of these deaths occurring in developing regions 70% in sub-Saharan Africa, and 16% in Southern Asia [1–3].To achieve the global goal of reducing maternal mortality to fewer than 70 deaths per 100,000 live births by 2030, an annual reduction rate of 11.6% is needed [4]. In Ethiopia, the maternal mortality rate was 412 per 100,000 live births in 2016, and efforts are ongoing to reduce this to 267 per 100,000 through key interventions like antenatal care, skilled birth services, and postnatal care [5, 6]. Currently, 48% of births in Ethiopia take place in health facilities [7].

Around 80% of global maternal deaths are due to direct causes like high blood pressure, infection, obstructed labor, hemorrhage, and unsafe abortions. An estimated 74% of these deaths could be prevented with proper access to interventions during pregnancy and childbirth [8]. It is generally recognized that a lack of access to, and inadequate utilization of, antenatal care (ANC) during pregnancy contributes to adverse maternal health outcomes such as maternal mortality [9].

In high-income and upper-middle-income countries, 99% of births are attended by skilled health personnel, compared to 68% in low-income and 78% in lower-middle-income countries [8].Skilled birth attendance (SBA) refers to childbirth that occurs in a healthcare facility, where a qualified healthcare professional, such as a doctor, midwife, or trained birth attendant assists the delivery [10].

Since the 1994 International Conference on Population and Development (ICPD) in Cairo, there has been a global focus on male involvement in sexual and reproductive health, shared responsibilities, and advancing gender equality. Key concerns highlighted include men’s active participation in family planning, pregnancy, and maternal and child healthcare [11, 12]. World Health Organization reported that the active involvement of men during pregnancy, childbirth, and the postpartum period as an effective strategy to improve maternal as well as newborn health outcomes in their 2015 recommendations on maternal and newborn health (MNH) promotion intervention [13]. Engagement of men/boys alongside women in gender-transformative programming is fundamental to addressing gender inequality and sexual and reproductive health and rights for all [14]. Increasing male involvement in maternal health is thought to be a promising and successful intervention for improving mother and newborn health outcomes [15].

A systematic review and meta-analysis of studies from low- and middle-income countries found that male partner involvement in prenatal care improves skilled birth attendance, institutional delivery, breastfeeding initiation, postpartum care, and maternal health outcomes, including birth preparedness and maternal nutrition [16–18].Previous primary studies done in the world show that the coverage of male involvement varies, ranging from 18% [19] to 77.3% [20]. Educational status [21–24], Employment [21], parity [21, 24], level of income [22], men’s level of knowledge [23, 25–27], distance [28, 29],age [28], marital status [28], , attitude health care workers [29],cost of delivery [30], positive attitude [20, 27],past experience of involvement [31] were predictors of male partner involvement in skilled delivery care service.

Previous systematic reviews and meta-analyses done on men’s involvement in maternal health in both developing and developed countries [17, 32–35], however, those studies have mainly focused on sexual reproductive health, family planning, and maternal health outcomes, yet there is a significant gap regarding male partner involvement in skilled delivery care service. Previous research done in Ethiopia has primarily addressed limited geographic areas, and small sample size, failing to account for national-level male participation in skilled delivery care service and its contributing factors in Ethiopia. To the best of our knowledge, this topic has not yet been investigated by systematic review and meta-analysis at the national level. Male partner involvement during childbirth is crucial for optimal health outcomes for women and children. To enhance maternal and infant health during the perinatal period, it is essential to understand the prevalence and factors contributing to male partner involvement in skilled birth care. Identifying these factors is critical for health stakeholders and policymakers in developing effective programs that improve the utilization of maternal, newborn, and child healthcare services in Ethiopia. This study aims to evaluate the pooled prevalence of male partner involvement in skilled delivery care service and the contributing factors in Ethiopia.

## Methods

### Study protocol and reporting

The Preferred Reporting Items for Systematic Reviews and Meta-Analyses (PRISMA) guidelines were followed to conduct this systematic review and meta-analysis [36](supplementary S1 file ). The eligibility criteria were adapted from the Newcastle Ottawa 2016 review guidelines [37].We used Endnote (version X7) reference management software to download, organize, and review and Zotero to cite related articles.

### Inclusion criteria

Study area: Ethiopia.

Study participants: The systematic review included all types of published quantitative studies on male partner involvement in skilled delivery care service.

Types of studies: Observational cross-sectional studies were included.

Outcomes of interest: The primary studies reported the prevalence of male partner involvement in skilled delivery care service and/or contributing factors.

Publication condition: Published articles were included in the form of journal articles without a time limit.

Language: American English.

Searching date of literature: We searched literature to conduct this systematic review and meta-analysis for a two-month duration (January 1, to February 30, 2024).

### Exclusion criteria

Articles without an abstract and/or full text, duplicate studies, anonymous reports, and qualitative studies were excluded from the analysis. In addition, studies that did not include outcomes in both the exposed and non-exposed groups were excluded after at least two email contacts with the primary author. These studies were excluded due to the inability to extract data from them in the absence of hard data. Furthermore, studies conducted in specific populations were excluded to make the studies included in the meta-analysis more similar concerning all important variables.

### Variables and measures

Skilled birth attendance is defined as a delivery that takes place at a medical facility staffed by a skilled birth attendant [10] .The place of residence was classified as rural or urban and men’s educational status was classified as secondary or above and below secondary, and level of knowledge was categorized as good or poor. The age of the partner is grouped into two categories: < 35 or = > 35 years. About favorable attitudes were grouped into positive and negative attitudes about skilled delivery care. About the previous experience of antenatal care is grouped as yes or no.

### Search strategy

We searched PubMed, Hinari, EMBASE, Cochrane, CINHAL, Google Scholar, and Mednar databases to identify relevant studies (supplementary S2 file). Initially, we searched PubMed, Google, and Google Scholar by article title to identify relevant key terms. Second, we identified similar ide keywords. Third, we searched the reference list of all identified reports and articles for additional studies and then searched the databases with these terms again. We used terms such as “male involvement, male participation’, ‘husband involvement’, ‘childbirth, ‘labor, ‘maternal health service’”, “husband accompaniment”, “male accompaniment”, “skilled birth care”, “associated factors”, “predictors”, “determinants”, “contributing factors”, “prevalence”, “magnitude”, “proportion”, “pregnant women” and “Ethiopia”. We tested and refined with multiple test searches, and similar search terms were combined using Boolean operators such as OR, while different concepts were combined using Boolean operators such as AND.

### Data extraction

MS Excel was used to extract the data. To gather the information required for analysis, two different data extraction formats were used. We included the last name of the author, the year of publication, the study region, the study design, sample size, the frequency of male involvement in skilled delivery care, the prevalence with its confidence interval, and the quality score of each study in the extraction form for prevalence. The data extraction format for contributing factors also contained the last name of the author and the publication year. Two authors independently gathered all essential data, then cross-checked them and reached a consensus on any differences.

### Quality assessment/critical appraisal

The searched article was manually exported into EndNote. Duplicates were removed, and the remaining articles were reviewed based on the inclusion and exclusion criteria. These criteria were tested on titles and abstracts to ensure their robustness in capturing articles related to male involvement in maternal skilled delivery care in Ethiopia. The Newcastle-Ottawa quality appraisal checklist was used to evaluate the quality of individual studies [37] (Supplementary S3 file).

Two reviewers independently assessed the quality of each primary study, and consensus was reached to either accept or reject each article based on the set criteria. When a disagreement occurred between the two reviewers, it was resolved by taking the mean score of the two reviewers. A study was considered to have a “low risk” if it was awarded > 50% of the quality assessment indicators.

All identified cross-sectional studies were appraised using eight items: inclusion criteria, description of study subjects and setting, valid and reliable measurement of exposure, objective and standard criteria used for the identification of confounders, strategies to handle confounders, outcome measurement, and appropriate statistical analysis. Finally, 10 studies and all were cross-sectional design, received a quality score of 50% or above on the quality scale, indicating that they are low risk and were included in the analysis.

### Statistical analysis

Stata statistical software (version 11.0, StataCorp. LP, College Station, United States of America) was used to perform all analyses. A random effects meta-analysis model based on the DerSimonian and Laird approach was used to pool the prevalence and identify contributing factors of male involvement in maternal skilled delivery care contact in Ethiopia. To determine statistical significance, a *p*-value and 95% confidence interval were used. The random effects model was used for analyses with statistical heterogeneity.

Statistical heterogeneity was checked using the I–squared (I2) statistic test [38]. The potential sources of heterogeneity were explored by meta-regression and subgroup analysis. Publication bias was viewed graphically by funnel plot asymmetry(Figure)and tested through Egger’s [39]. The *p*-value was > 0.05; there was statistical evidence for the absence of publication bias using the Egger test.

## Result

A total of 850 studies (PubMed = 95, Hinari = 10, Cochrane Review = 85 EMBASE = 20, Google Scholar = 640) published articles were identified. Of all the articles, 200 were removed due to duplicates. Based on the inclusion and exclusion criteria of title and abstract selection, the eligibility of 650 abstracts was evaluated. The articles that did not fulfill the criteria were removed (*n* = 580), leaving a total of 70 articles for full-text screening.

**Fig. 1:**
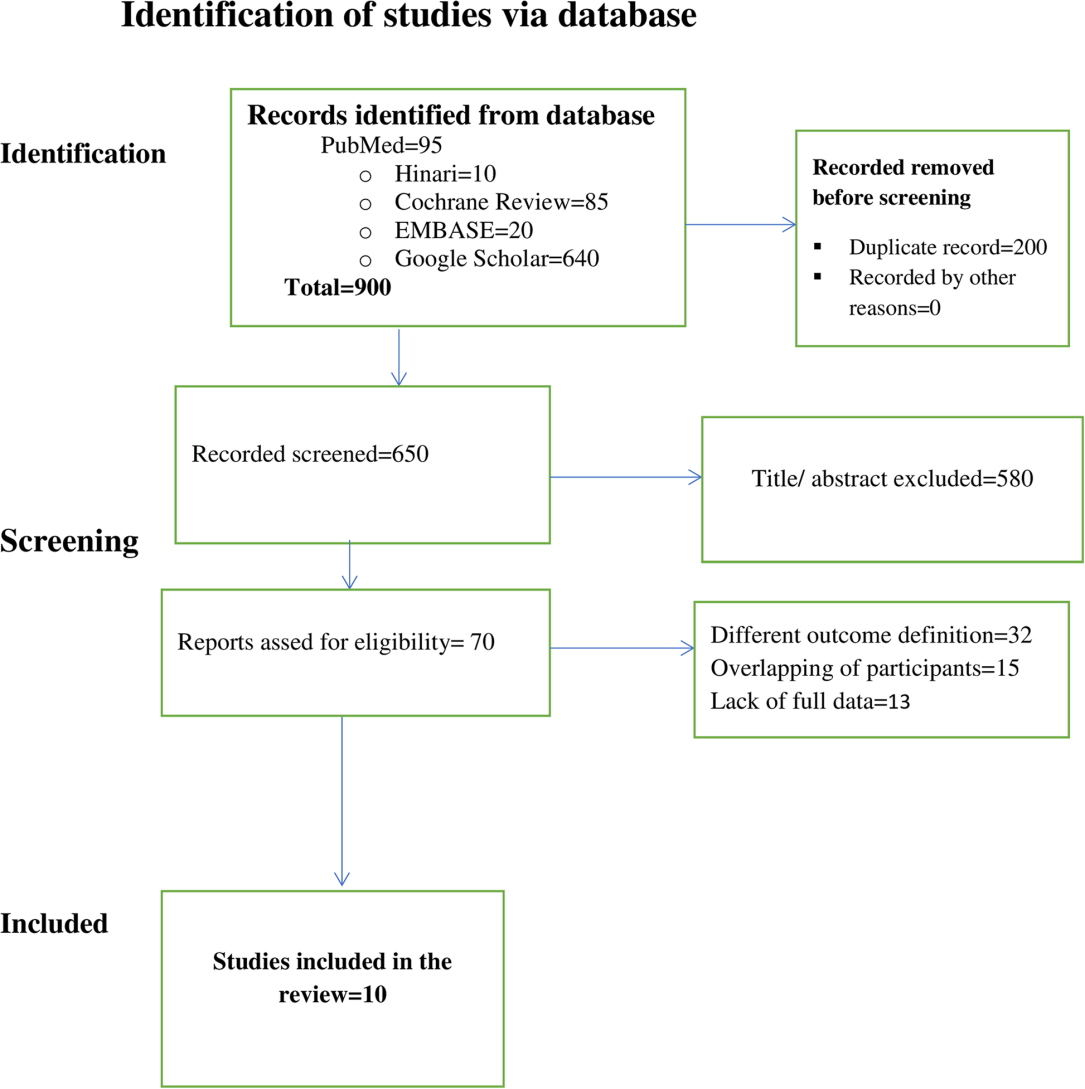
PRISMA flowchart diagram of the study selection process

A total of 70 articles were screened according to the eligibility criteria for full-text selection. Furthermore, 60 articles were excluded due to researchers reporting different outcomes of interest, overlapping study participants, poor methodological quality and a lack of full data. Finally, 10 studies were included in the meta-analysis. The same data source was used to prevent participant overlap and one or more publications were eliminated based on the studies’ overall quality scores, with the highest-scoring research being included (Fig. 1).

### Study characteristics

The final 10 studies were included in our analysis [40–49]. They included a study population of 5307 men, of whom 2770 males attended during maternal skilled delivery care. Among the included studies published articles were included.

From the included studies all studies were cross-sectional by design with 9 studies in a community setting and 1 study included a facility-based study that reported male involvement on skilled birth care.The sample sizes across the studies ranged from335 [49] to 767 [47] (Table 1).

**Table 1:** Description of included variable in meta-analysis

Id no	Author	Year	Study Design	Region	Actual Sample	Frequency	ES**[95%C]
1	Mickiale Hailu, et al.	2023	Cross-sectional	Oromia	603	312	51.8(47.8–55.8)
2	Kassanesh Melese Tessema, et al.	2019	Cross-sectional	Amhara	477	181	37.9(33.6–42.3)
3	Zerihun Tamirat, et al.	2015	Cross-sectional	SNNP	635	262	41.3(37.5–45.1)
4	Katiso NA, et al.	2014	Cross-sectional	SNNP	335	128	38.2(33–43.4)
5	Meseret Alemu, et al.	2023	Cross-sectional	SNNP	583	251	43.1(39.1–47.1)
6	Shewangizaw Hailemariam, et al.	2021	Cross-sectional	SNNP	767	280	36.5(33.1–39.9)
7	Bikila Lencha Gemechu, et al.	2023	Cross-sectional	Oromia	701	518	73.9(70.6–77.1)
8	Abdusamed Mohammed, et al.	2022	Cross-sectional	Oromia	399	336	84.2(80.6–87.8)
9	Destaw, et al.	2014	Cross-sectional	Addis Abeba	422	341	80.8(77.0–84.6)
10	Daniel Belema Fekene, et al.	2019	Cross-sectional	Oromia	385	161	52.9(40.6–65.3)

### Pooled prevalence of male involvement in skilled delivery care in Ethiopia

**Fig. 2:**
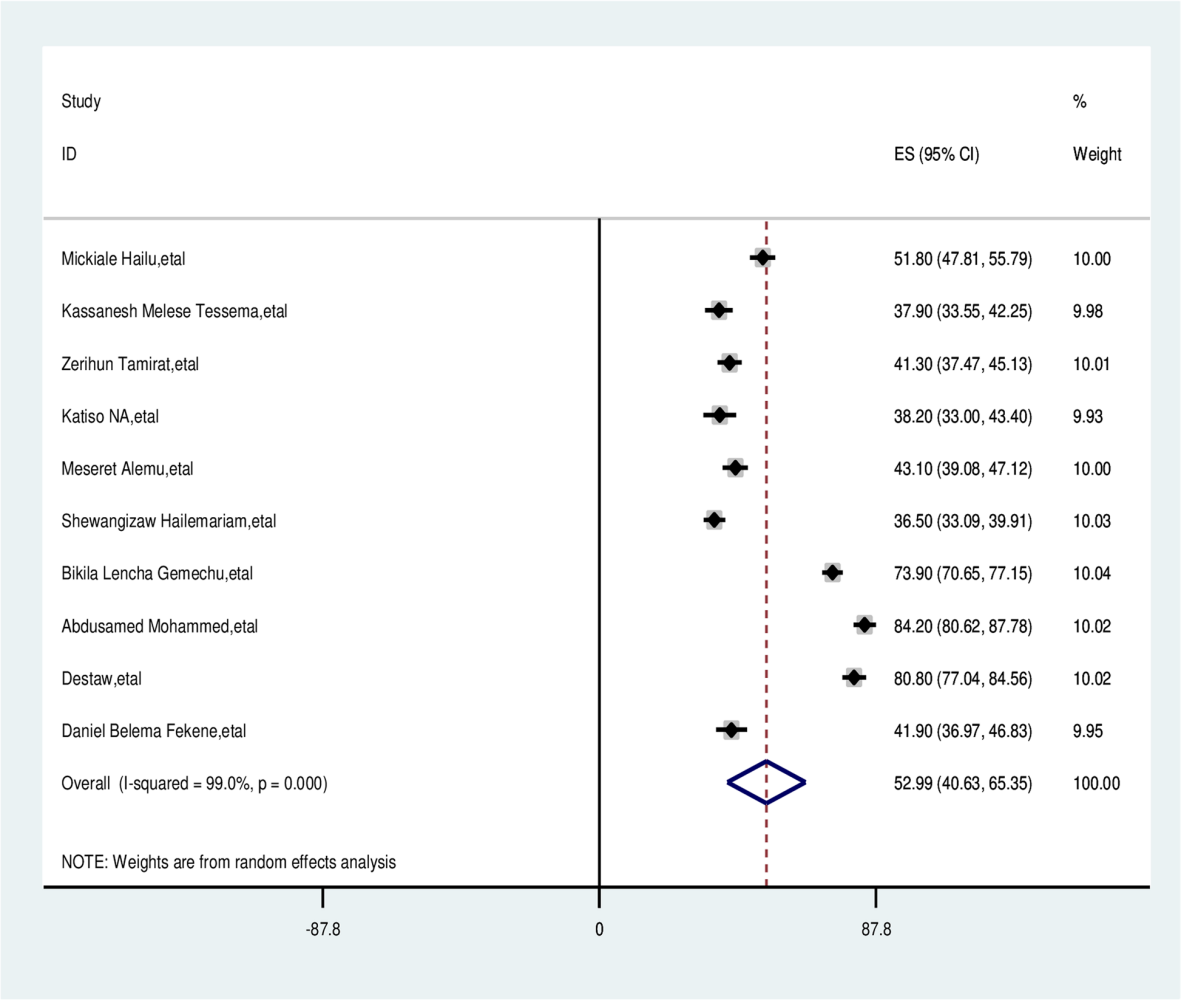
Overall pooled prevalence of male partner involvement in skilled delivery care service in Ethiopia, 2024

The overall pooled prevalence of male partner involvement in skilled delivery care was 52.99% [95% CI: 40.63–65.35]. Using the random effects model, the pooled effect size of male involvement in skilled delivery care showed statistically significant heterogeneity among the included studies (I2 = 99.0%, *P* = 0.00) (Fig. 2).

### Subgroup analysis

We performed a subgroup analysis by year to address heterogeneity. The subgroup analysis showed that the prevalence of male involvement in maternal skilled delivery care ranged from 37.9% in 2019 to 84.2% in 2022. Substantial heterogeneity was estimated, at up to 99.4% in 2014 (Table 2).

**Table 2:** Subgroup analysis of the pooled prevalence of male involvement in maternal skilled delivery care in Ethiopia 2024

Variable	Characteristics	No of study	Pooled prevalence	I2	*P* value
Time(year)	2018	2	36.67	89.8%	0.002
	2016	1	59.9	-	-
	2022	2	47.5	97.9%	0.00
	2014	2	42.5	99.4%	0.00
	2019	1	60	-	-
	2017	1	24	-	-
	2020	1	62	-	-

### Publication bias

**Fig. 3:**
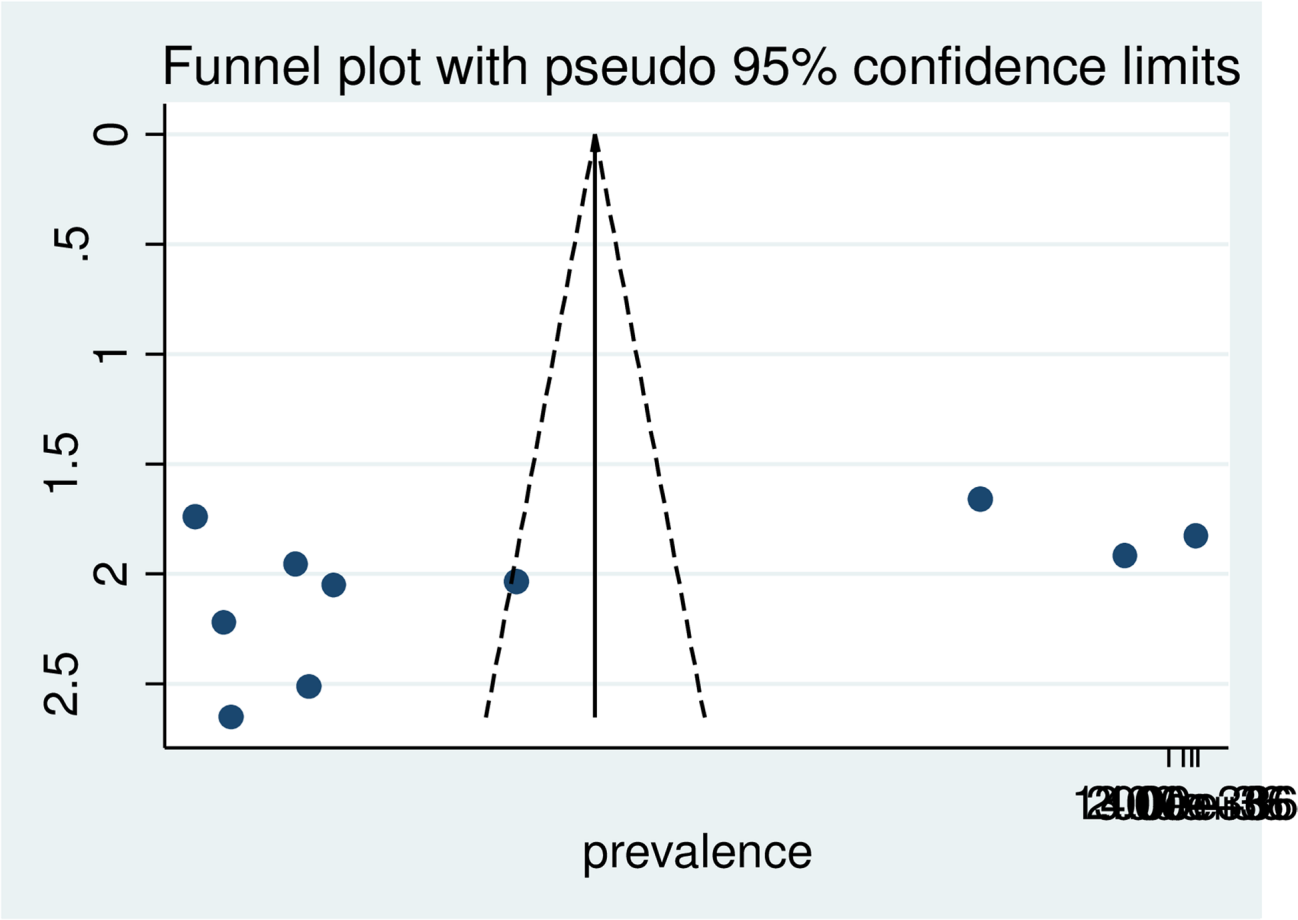
Funnel plot publication bias plot for the prevalence of male partner involvement in skilled delivery care in Ethiopia

Publication bias was viewed graphically by funnel plot asymmetry (Fig. 3) and tested through Egger’s [39]. The *p*-value was > 0.05; there was statistical evidence for the absence of publication bias using the Egger test. Egger’s regression test was not statistically significant, with a P value of 0.161.

### Sensitivity analysis

In this meta-analysis, no single study dominated the pooled prevalence of male partner involvement in skilled delivery care service in Ethiopia, according to the results of a random-effects model (Table 3).

**Table 3:** Shows a sensitivity analysis of male partner involvement in skilled delivery care service in Ethiopia

Study omitted Estimate [95% Conf. Interval]
Mickiale Hailu, et al. 53.120052 39.344757 66.895348
Kassanesh Melese Tessema, et al. 54.664669 41.521191 67.808144
Zerihun Tamirat, et al. 54.290169 40.892555 67.687782
Katiso NA, et al. 54.621952 41.483665 67.760239
Meseret Alemu, et al. 54.088398 40.614838 67.561958
Shewangizaw Hailemariam, et al. 54.830921 41.892529 67.769318
Bikila Lencha Gemechu, et al. 50.658028 37.670006 63.646053
Abdusamed Mohammed, et al. 49.520714 37.932274 61.10915
Destaw, et al. 49.89946 37.607822 62.191101
Daniel Belema Fekene, et al. 54.215668 40.918381 67.512955
**Combined 52.991279 40.629757 65.352802**

### Determinants of male partner involvement in skilled delivery care service

We included six selected variables to identify relationships with male involvement in skilled delivery care in Ethiopia. Of these, five variables, namely level of knowledge regarding SBA, paternal attitude, ANC accompanying experience, urban residence, and educational status of men, were identified as statistically significant contributing factors to male partner involvement in institutional delivery care in Ethiopia. The review also demonstrated that paternal age had no statistically significant association with male involvement in facility delivery care (Table 4).

**Table 4:** Factors associated with male partner involvement in skilled delivery care service in Ethiopia

Variable	Exposed	Comparator	OR (95% CI)	I 2	Egger test
Paternal level of knowledge	Good knowledge	Poor knowledge	3.129;1.90, 4.35.	99.5%	0.794
Paternal Educational status	Secondary and above	Illiterate/low education	1.994;1.69,2.30	88.1%	0.567
Residence	Urban	Rural	2.12; 1.61,2.64	93.7%	0.063
Attitude	Positive	Negative	2.39; 1.45,3.34,	89.2%	0.974
ANC accompanying experience	Accompanying ANC	Not accompanying ANC	8.09;3.14,19.32	99.9%	0.325
Paternal age	< 35 years	=>35 years	2.40;0.72, 4.27	91%	0.017

### Level of knowledge

The overall analysis of studies showed that knowledge of ANC is associated with male involvement in skilled delivery care. Men with good knowledge were 3 times more likely to attend their ANC booking compared to men with poor knowledge (OR, 3.129; 95% CI: 1.901, 4.356). A random effects model was assumed for the analysis as I2 (99.5%) and Egger test 0.794 with a *p*-value of (< 0.001) showed statistically significant heterogeneity among the included studies for this factor analysis.

### Paternal educational status

The overall analysis of studies showed that knowledge of ANC is associated with male involvement in skilled delivery care. Men with good knowledge were three times more likely to attend their ANC booking compared to men with poor knowledge (OR, 1.994 95% CI: 1.688 2.300). A random effects model was assumed for the analysis as I2 (88.1%) and Egger test 0.567 with a *p*-value of (< 0.001) showed statistically significant heterogeneity among the included studies for this factor analysis.

### Partner attitude

The meta-analysis showed that paternal positive attitude was significantly associated with male involvement in skilled delivery care. The overall odds ratio was 2.39 with a 95% CI of 1.45–3.34, and a *p*-value of < 0.001, indicating that men with a positive attitude were 2.4 times more likely to attend skilled delivery care compared to their counterparts. A random effects model was used for the analysis, showing statistically significant heterogeneity among the included studies (I2 = 89.2, Egger test 0.325, *p*-value < 0.001).

### ANC accompanying experience

Paternal ANC experience was 8 times more likely to attend skilled delivery care than those who had no ANC accompanying experience (OR 8.09, 95% CI 3.14–19.32).

A random effects model was assumed for the analysis as I2 was 99.9% and the Egger test showed a *p*-value of < 0.001, indicating statistically significant heterogeneity among the included studies.

### Residence

Residence was associated with male involvement in skilled birth care. Urban residents were 2 times more likely to be involved in skilled delivery care than rural residents (OR 2.12, CI 1.61–2.64). A random effect model was used for the analysis with I2 = 93.7%, Egger test was 0.063 and *p*-value = 0.001 with statistically significant heterogeneity among the included studies.

### Paternal age

The overall analysis of studies showed that paternal age has no association with male involvement in skilled delivery care. Paternal age < 35 years showed no significant association compared to age > 35 years (OR, 2.40; 95% CI: 0.72, 4.27). A random effects model was assumed for the analysis as I2 (91.0%), Egger test 0.017 with a *p*-value (< 0.001) showed statistically significant heterogeneity and publication bias among the included studies for this factor analysis.

## Discussion

This study, to the best of our knowledge, is the first to examine the impact of male involvement during facility delivery care in Ethiopia. We build on previous research by incorporating a composite measure of male involvement in institutional delivery care and its contributing factors. Most studies examining the impact of men’s involvement on maternal or reproductive health have produced general results. However, this analysis focuses on specific outcome variables and uses a single geographically diverse sample. The study thoroughly analyzes the national coverage of male partner involvement in maternal skilled delivery care services. The pooled overall prevalence of male partner involvement in skilled delivery care service was 52.99% [95% CI: 40.63–65.35].

The proportion of studies on maternal facility delivery care coverage that included male participants varied from 18% [19] to 77.3% [20]. There are several possible reasons for this discrepancy, such as the amount of research done, regional variations in culture and socioeconomic status, unequal access to maternity healthcare facilities, and the publication date of the article. This article is in line with a study done in Africa 45.7% [50], in Nigeria,51.1% [51], in Indonesia,41.2% [52],in Tanzania(54.4%) [53], in Kenya (54.1) [54], in Mbeya 56.9% [55]and inGhana,44.5% [56].This might be because, in many low- and middle-income countries, traditional gender roles limit male involvement in reproductive health, as childbirth is viewed as a woman’s responsibility. However, higher male involvement is linked to better paternal education, socioeconomic status, and healthcare access. Shared challenges like limited healthcare infrastructure and public health campaigns in Africa and Asia have contributed to similar male involvement rates in skilled delivery care service.

However, it was lower than that of a study conducted, in Ghana (67.2%) [57], in Uganda 77.8% [58], in Myanmar,87% [59], and in Nigeria 80.4% [60]. The lower male involvement in Ethiopia compared to studies in Ghana, Uganda, Myanmar, and Nigeria may be due to several factors. These countries may have stronger public health campaigns, better healthcare infrastructure, or greater societal acceptance of male involvement in maternal health. Additionally, cultural differences, variations in paternal education, and accessibility to health services might contribute to the higher involvement rates in these countries. This review shows better results compared to Kangundo Sub-County Hospital in Kenya(34.1%) [61]and kenya18% [54]. The higher male involvement in Ethiopia compared to the studies in Kenya could be attributed to several factors. Ethiopia may have more targeted public health campaigns promoting male participation in maternal care [62], or there might be differences in cultural norms and attitudes toward male involvement in healthcare decisions. Additionally, Ethiopia could have better access to healthcare services or educational initiatives that raise awareness of the importance of male involvement in reproductive health.

In this finding, namely level of knowledge regarding SBA, paternal attitude, ANC accompanying experience, urban residence, and educational status of men, were identified as statistically significant contributing factors to male partner involvement in skilled delivery care service in Ethiopia.

The finding is that good knowledge is associated with better male partner involvement in skilled delivery care services than poor knowledge. This finding was similar to evidence in Indonesia [52], in Buyende District, Uganda [58] and inNigeria [60, 63]. The justification can be explained by the fact that increased knowledge often leads to greater awareness of the importance of maternal health and the benefits of skilled care during childbirth. Men with more knowledge are likely to understand the risks of unassisted deliveries and the advantages of seeking professional care. Additionally, knowledgeable partners may be more supportive and involved in ensuring the well-being of both mother and child during delivery. They also know about the danger signs and complications of childbirth, which helps to reduce the fear of male involvement in facility delivery care and encourages them to take greater responsibility for the health of their partners and their unborn children [64].

The finding is that men with secondary education and above are more likely to be involved in maternal facility delivery care service than their counterparts.This figure is congurent to study done in Ghana [57, 65, 66],in Nigeria [60],in Centeral Nigeria [67] and in Kenya [68]. Educated men may have better access to health-related information, leading to greater awareness of the importance of maternal healthcare services [69]. Education often correlates with an improved understanding of the risks of home births and the benefits of skilled birth attendance. Additionally, higher education levels can result in more progressive views on gender roles, encouraging men to actively participate in reproductive health matters. Men are better equipped to evaluate and comprehend the risks of pregnancy for both the mother and the fetus when they have received education. Education also enables men to discard negative attitudes and cultural beliefs [64]. It is also likely that men with a high level of education have formal employment, which enables them to raise funds that they can use to pay for hospital bills for delivery services.

The review also found that male partners who had a positive attitude were 2.39 times more likely to be involved in institutional delivery care than their counterparts. This is similar to a study done in Bangladesh [70], in Pakistan [71], in Sub-Saharan Africa and [72]and in India [73]. A positive attitude often reflects greater awareness and understanding of the importance of skilled birth attendance, which can motivate men to support institutional deliveries [74]. Men with supportive attitudes may also be more willing to challenge traditional gender roles that view childbirth as solely a woman’s responsibility. Furthermore, positive attitudes are often linked to higher levels of education, exposure to health promotion campaigns, and access to information, all of which encourage active participation in maternal health services.

Moreover, this review found that paternal accompaniment to ANC increased participation in facility birth care by 8 times compared to counterparts. This figure is similar to a study done in Bangladesh [75]. This can be justified by the fact that when men accompany their partners to ANC visits, they become more informed about maternal health and the importance of skilled birth attendance. Such involvement helps men understand the risks of home deliveries and encourages them to support facility-based births. Additionally, attending ANC together fosters shared decision-making, making men more invested in ensuring a safe delivery, and knowing about pregnancy-related complications and danger signs during pregnancy and childbirth, creating more awareness during the antenatal care service period, thus leading to greater male participation in facility birth care.

Additionally, this study found that urban men were twice as likely to be involved in institutional birth care as rural men, a finding consistent with studies conducted in Bangladesh [76] and India [77]. This can be attributed to better access to healthcare facilities, greater exposure to health education, and more progressive attitudes toward maternal health in urban areas compared to rural regions [78]. Urban men may also have higher levels of education and socioeconomic status, factors that typically increase awareness and engagement in institutional birth care [79]. It can also be argued that men had better communication with their wives, better-coping abilities, negotiating skills, and better participation in their wives’ skilled birth care services.

## Limitations of the study

The high I² values indicate substantial heterogeneity across the included studies, making it difficult to generalize the pooled results. With only 10 cross-sectional studies, the meta-analysis may lack sufficient statistical power to detect small effects or fully explore subgroup differences, and the design limits the ability to infer causality between factors and male involvement in delivery care.

## Conclusion and recommendation

This study found that more than half of male partners in Ethiopia were involved in delivery care services. Key factors positively associated with male involvement included higher paternal education, better knowledge, a positive attitude towards maternal health, previous ANC accompaniment experience, and urban residence. These findings emphasize the need for targeted interventions and awareness campaigns that address these specific factors to enhance male participation in maternal health. Promoting male involvement in reproductive health services could significantly improve maternal and child health outcomes in Ethiopia. Future research should incorporate qualitative methods to gain deeper insights into the attitudes and perceptions of men, women, and healthcare workers regarding male involvement in maternal health.

## Supplementary Information

Supplementary Material 1. The PRISMA checklist dox.

Supplementary Material 2. Searching strategy dox.

Supplementary Material 3. Newcastle-Ottawa Quality Assessment Scale for cross-sectional studies used in the systematic review and meta-analysis 2024.

## Data Availability

The datasets used and analyzed during the current study are available from the manuscript and supplementary material.
