# Supplementary material for: Male partner involvement in delivery care service and associated factors in Ethiopia: a systematic review and meta-analysis
Source: BMC Health Serv Res. 2024 Nov 26;24:1467. doi: 10.1186/s12913-024-11993-y (PMC11590235; doi:10.1186/s12913-024-11993-y)
Supplement: Supplementary file 2 — Supplementary Material 2. Searching strategy dox. [file 12913_2024_11993_MOESM2_ESM.docx]

| Google scholar | | | | Result |
| --- | --- | --- | --- | --- |
|  | Concept | | **Search detail** |  |
| Concept1 map | Male partner involvement in Skilled delivery care service | Keyword |  |  |
|  |  |  | "skilled delivery care service"[tw] OR "male involvement"[ tw] OR "male involvement in skilled delivery care service"[tw] OR “child birth” [tw] OR “ | 892 |
|  |  | Mesh term | “skilled delivery care service” [Mesh] | 1,750 |
| Concept 3 map | Associated factors | Keyword | "Associated factors" [tw] OR "determinants"[tw] OR "Predictors"[tw] OR "Factors contributing"[tw] OR "correlates"[tw] | 3,302 |
| Concept 3 map | Male partner involvement Postpartum women | Keyword | "child birth"[tw] “postnatal women” [tw] | 457 |
|  |  | Mesh term | "postpartum w"[MeSH Terms] | 568 |
| Concept 4 map | Ethiopia | Keyword | Ethiopia [tw] | 371 |
|  |  | Mesh term | "Ethiopia "[MeSH Terms] | 23 |
| Concept 5 map | Prevalence | Keywords | Prevalence[tw] or Magnitude[tw] OR Proportion[tw] | 2,367 |
|  |  | Mesh term | "Prevalence"[Mesh] | 456 |
| ((“We used terms such as “male involvement, male participation’, ‘husband involvement’, ‘childbirth, ‘labor, ‘maternal health service’”, “husband accompaniment”, “male accompaniment”, “skilled birth care”, “associated factors”, “predictors”, “determinants”, “contributing factors”, “prevalence”, “magnitude”, “proportion”, “pregnant women” and “Ethiopia”. We tested and refined with multiple test searches, and similar search terms were combined using Boolean operators such as OR, while different concepts were combined using Boolean operators such as AND)). | | | | 504 |
| PubMed | | | |  |
| Male partner involvement in skilled delivery care service in Ethiopia | | | | 324 |
| Hinari | | | |  |
| Male partner involvement in skilled delivery care service in Ethiopia | | | | 76 |
| Other data base sources | | | | 53 |
