# Supplementary material for: Male partner involvement in delivery care service and associated factors in Ethiopia: a systematic review and meta-analysis
Source: BMC Health Serv Res. 2024 Nov 26;24:1467. doi: 10.1186/s12913-024-11993-y (PMC11590235; doi:10.1186/s12913-024-11993-y)
Supplement: Supplementary file 3 — Supplementary Material 3. Newcastle-Ottawa Quality Assessment Scale for cross-sectional studies used in the systematic review and meta-analysis 2024. [file 12913_2024_11993_MOESM3_ESM.docx]

**Supplementary S 3 file:** Newcastle-Ottawa Quality Assessment Scale for cross sectional studies used in the systematic review and meta-analysis 2024.

|  | **Selection** | | | | **Comparability** | **Outcome** | | **Total score** |
| --- | --- | --- | --- | --- | --- | --- | --- | --- |
| Authors | Representativeness s (1) | Sample size (1) | Non respondents (1) | Ascertainment of the exposure (risk factor) (2) | The subjects in different outcome groups are comparable, based on the study design or analysis. confounding factors are controlled (1) | Assessment of the outcome (2) | Statistical test (1) |  |
| Mickiale Hailu, etal | 1 | 1 | 1 | 0 | 1 | 2 | 1 | 7 |
| Kassanesh Melese Tessema, etal | 1 | 1 | 1 | 1 | 1 | 2 | 1 | 8 |
| Zerihun Tamirat, etal | 1 | 1 | 1 | 2 | 1 | 2 | 1 | 9 |
| Katiso NA, etal | 1 | 1 | 1 | 2 | 1 | 1 | 1 | 8 |
| Meseret Alemu, etal | 1 | 1 | 1 | 0 | 1 | 2 | 1 | 7 |
| Shewangizaw Hailemariam, etal | 1 | 1 | 1 | 2 | 1 | 2 | 1 | 9 |
| Bikila Lencha Gemechu, etal | 1 | 1 | 1 | 1 | 0 | 2 | 1 | 7 |
| Abdusamed Mohammed, etal | 1 | 1 | 1 | 1 | 1 | 1 | 1 | 7 |
| Destaw, etal | 1 | 1 | 1 | 1 | 1 | 2 | 1 | 8 |
| Daniel Belema Fekene, etal | 1 | 1 | 1 | 1 | 1 | 2 | 1 | 8 |
